# The Effect of Chronic Disease and Mental Health on Sleep Quality among Migrant Elderly Following Children in Weifang City, China

**DOI:** 10.3390/ijerph191912734

**Published:** 2022-10-05

**Authors:** Jieru Wang, Wen Qin, Mingli Pang, Bo Zhao, Jing Xu, Shixue Li, Fanlei Kong

**Affiliations:** 1 Centre for Health Management and Policy Research, School of Public Health, Cheeloo College of Medicine, Shandong University, Jinan 250012, China; 2 NHC Key Lab of Health Economics and Policy Research, Shandong University, Jinan 250012, China; 3 Shandong University Hospital, Cheeloo College of Medicine, Shandong University, Jinan 250012, China; 4 Department of Health Administration, Graduate School, Yonsei University, 1 Yonseidae-gil, Wonju 26493, Korea

**Keywords:** sleep quality, chronic disease, mental health, depression, anxiety, migrant elderly following children

## Abstract

Few studies have focused on the sleep quality among migrant elderly following children (MEFC). This study aimed to investigate the effects of chronic disease and mental health on the sleep quality of MEFC in Weifang, China. A cross-sectional study was conducted by multi-stage cluster random sampling, and in total 613 participants were enrolled. Sleep quality and mental health were assessed by the Pittsburgh Sleep Quality Index (PSQI) and the Depression, Anxiety, and Stress Scale (DASS-21), respectively. Chronic disease was assessed by the question “how many chronic diseases do you have?” A descriptive analysis and chi-square test were used to describe participants’ sociodemographic variables, chronic disease, mental health, and sleep quality. The relationship between chronic disease, mental health, and sleep quality was explored by establishing binary logistic regression models. The results showed that 18.3% of MEFC’s sleep quality was poor. MEFCs who were male were more likely to report good sleep quality. MEFCs who have similar monthly family income compared with others around, with multimorbidity, depression, and anxiety were more likely to report poor sleep quality. Nearly 1/5 of MEFCs were having poor sleep quality in this study. Results indicated that chronic diseases, depression, and anxiety were risk factors for the sleep quality of MEFC. Implications for the government, communities, and families of MEFC were given for improving their sleep quality.

## 1. Introduction

China ranks first with a total of 1.44 billion people, becoming the country with the largest population in the world (18%). Among them, the proportion of the population aged 60 and above was 264 million, accounting for 18.70% of the total Chinese population, and has increased by 5.44% since 2010 [1,2]. In addition, China’s reform and opening not only brought huge economic benefits but also a serious regional economic development imbalance [3], due to the different geographical conditions. Therefore, many people chose to migrate to big cities for a better life. China’s official data (2015, National Health and Family Planning Commission Survey) showed that migrating older individuals shared 7.2% of the total migration population, about 80% of whom are under 70 years old [4]. In this study, those migrant older people who left their native place and then migrated following their children to big cities to take care of children, retire, and provide for the elderly were referred to as the migrant elderly following children (MEFC) [5].

Good sleep quality is essential for improving health and health-related quality of life. Because sleep is restful and restorative, sleep quality is also a public health concern [6]. The World Health Organization said sleep is a basic human need that is critical for good health and daytime performance [7]. Some studies have pointed out that without enough good sleep, the brain struggles to perform basic functions, making people harder to concentrate or causing cognitive decline that makes it harder to remember things [8,9]. However, previous studies showed that sleep problems were prevalent among older adults [10,11,12,13]. A study based on demographic and cross-sectional survey found that the overall prevalence rates of poor sleep quality were 33.8% among rural older adults in Shandong Province, China [14]. A prospective cohort study conducted in Japan demonstrated that 28.9% of older adults had poor sleep quality [15]. Moreover, a previous study also pointed out that the sleep quality of migrants needs to be improved [16]. A study among older African American adults showed that 71.0% of the participants’ sleep quality was poor [17]. These results indicated the sleep quality of older adults, especially migrant elderly, should be paid more attention.

A variety of factors contribute to poor sleep quality. Disease or illness is one of the most important factors affecting healthy sleep. Existing studies found that chronic diseases were prevalent among older adults [18,19,20]. Of the people over 60 years old, 75.8% had one or more chronic diseases in China [21], and 7.39% of rural Chinese migrants had chronic diseases (hypertension or diabetes) [22]. Additionally, previous studies have shown that the total number of chronic diseases is one risk factor for sleep quality in older adults [23,24]. Among middle-aged and older individuals, the number of chronic conditions was significantly associated with poor sleep quality [25]. Zhang et al. conducted a cross-sectional study in Guangdong Province, China, and found that middle-aged people with two or more chronic diseases were almost nine times more likely to have poor sleep quality [26]. Another cross-sectional study also identified self-reported chronic disease as one of the major factors affecting the rates of sleep deprivation among migrant populations aged 18–59 years old in China [27]. Therefore, it is important to study the relationship between chronic diseases and sleep quality among the migrant elderly. Based on the above, the hypothesis is thus proposed that chronic disease is negatively correlated with sleep quality among MEFCs.

Mental health is another important factor in determining sleep quality in older adults. Yet the World Health Organization has pointed out that approximately 15% of adults aged 60 or over suffer from a mental disorder [28], and an estimated 54 million people in China suffer from depression, and about 41 million suffer from anxiety disorders [29]. Some existing studies have found that better mental health (for example, lower anxiety and depression) improves the quality of life and sleep in older adults [30,31]. The findings of Fu et al. suggested that perceived stress was a risk factor for sleep quality among Chinese older adults [32]. Another study conducted by Wang also demonstrated that both depression and anxiety negatively affected sleep quality among older adults in Jinan, Shandong Province, China [33]. Additionally, a cross-sectional study conducted in Singapore revealed that depression and anxiety were significantly correlated with sleep quality in Chinese people [34]. The rate of sleep deprivation was 1.46 times and 1.33 times higher in migrant groups aged 18–59 with high work and life pressures than those without work and life pressures in China [27]. The results for Macau migrants also showed that mental health affected sleep quality [35]. Therefore, in MEFC, the relationship between mental health, especially depression and anxiety, and sleep quality is also worth exploring. Based on the previous results, the hypothesis is proposed that mental health is negatively correlated with sleep quality among MEFCs.

Given the lack of research on the sleep quality status of Chinese MEFC and the impact of chronic diseases and mental health on it, this study aimed to investigate the sleep quality of MEFC and further explore the association between chronic disease, mental health, and sleep quality among MEFC in Weifang, China.

## 2. Materials and Methods

A cross-sectional study was conducted in August 2021 in Weifang City, Shandong Province, China. According to the results of the Seventh National Census, there was a 9.39 million resident population in Weifang City. Among them, the population aged 60 and above was 2.04 million (accounting for 21.77%), including 1.48 million people aged 65 and above [36].

This study used multi-stage cluster random sampling to enroll participants. Firstly, 4 districts in Weifang were selected. Secondly, 4 streets were randomly chosen from the first stage. Lastly, 4 communities were also selected randomly from each sub-sampling unit. All older migrant adults in these communities who migrated to Weifang following their children consisted of the total sample for the research. The following criteria were used to include participants: (1) people aged 60 or above, (2) household registration beyond Weifang, and (3) the ability to communicate with surveyors smoothly.

Twenty-five college students comprised the investigators in this study. These investigators had been trained on the research background, questionnaire contents, and social survey skills before the questionnaire survey. The data were collected through face-to-face interviews with participants. A total of 613 participants were finally included in the data analysis.

### 2.1. Measurement

#### 2.1.1. Sociodemographic Characteristics

The sociodemographic characteristics included sex, age (How old are you?), hukou (Are you from an urban or rural area?), marriage status (Are you married?), education level (What is your education level?), BMI (What is your height and body weight?), working status (What is your working status?), migration willing (How about your migration willing?), migration reason (What is your migration reason?), source of living expenses (What is your source of living expenses?), and monthly household income compared with others around (What is your feeling in comparison monthly household income with others around?).

Sociodemographic characteristics were divided into sub-categories as follows: sex (Male, Female); age (60–69 years old, 70–79 years old, 80 years old or above); hukou (Rural, Urban); marriage status (Married, Single); educational level (Illiterate, Primary school, Junior high school, High school and above); BMI (Underweight, Normal, Overweight, Obesity) [37]; working status (Employment, Retired, Unemployment); migration willing (Unwilling, Fair, Willing); migration reason (Taking care of grandchildren, Not taking care of grandchildren); source of living expenses (Own, Spouse and children, Others); and monthly household income compared with others around (Higher, Similar, Lower).

#### 2.1.2. Chronic Disease Status

Chronic disease status was measured by the question “Do you have any chronic diseases?” If participants answered yes, the following question, “How many chronic diseases do you have?” would be asked. A previous study had defined people with two or more chronic diseases as multimorbid [38]. Therefore, the chronic diseases in this study included: zero, one chronic disease, and multimorbidity (two or more chronic diseases).

#### 2.1.3. Mental Health

The Depression Anxiety Stress Scale (DASS-21) was employed to evaluate the negative states of three mental health conditions of participants: depression, anxiety, and stress [39]. DASS-21 was a short form of the DASS, which was a self-report 4-point Likert scale that consisted of 21 items. Participants were asked for each item, and responses ranged from 0 to 3 (“0” for “not at all”, “1” for “not very”, “2” for “somewhat”, and “3” for “very”). Each subscale consisted of 7 items. The scores obtained on each of the three subscales of DASS-21 were summed and multiplied by 2; therefore, the sum scores of the three subscales ranged from 0–42 and the total score of DASS-21 ranged from 0 to 126. The sum scores of 0–9 for depression, 0–7 for anxiety, and 0–14 for stress were considered normal, while the sum score of 10–42 for depression, 8–42 for anxiety, and 15–42 for stress were considered as depressed, anxious, and stressed [40]. DASS-21 has been proven to be a reliable and effective measure for assessing the mental health of the Chinese population [41,42].

#### 2.1.4. Sleep Quality

The Pittsburgh Sleep Quality Index (PSQI) was a self-rated questionnaire that was used to evaluate the sleep quality of the participants in the last month [43]. The PSQI consisted of 19 questions and was divided into 7 components, including subjective sleep quality, sleep latency, sleep duration, habitual sleep efficiency, sleep disturbances, use of sleeping medication, and daytime dysfunction. Each component was weighted equally on a 0 to 3 scale and the total score ranged from 0 to 2l. A higher score meant worse sleep quality, while a score >7 indicated poor sleep quality [44]. PSQI has been proven to be a reliable and effective measure for assessing the sleep quality of the Chinese population [45,46].

### 2.2. Statistical Analysis

All statistical analyses were performed with SPSS 24.0, and *p* values < 0.05 were considered a statistically significant difference. Firstly, descriptive analysis and chi-square tests were used to explore the difference between sociodemographic variables, chronic disease, mental health, and sleep quality. Secondly, a binary logistic regression analysis was conducted since sleep quality in this study was a dichotomous variable. These 3 models were developed while odd ratios (OR) and 95% confidence intervals (95% CI) were calculated.

## 3. Results

### 3.1. Participants’ Characteristic

Table 1 showed the sociodemographic information of MEFC. According to Table 1, the majority of participants were aged 60–69 years old (79.3%), 73.1% were female, 87.9% were married, and most were from a rural area (85.6%). In terms of education level, 17.8% of MEFC were in high school and above, while more than half were in primary school or below (56.5%). Moreover, 47.3% of MEFC were overweight and 11.1% were obese. In addition, 83.5% of participants were willing to migrate with children, and 86.9% migrated to take care of grandchildren. The main source of the living expense was from their spouse and children (64.1%), while 10.8% of MEFC thought their monthly household income was higher than the surrounding area.

Table 1 also showed that sex, marital status, source of living expenses, and monthly household income compared with others were significantly correlated with the sleep quality of MEFC.

### 3.2. Chronic Disease and Mental Health with Sleep Quality

Table 2 showed the participants’ depression, anxiety, stress, chronic disease, and sleep quality. In terms of chronic disease, 27.7% of MEFCs have one chronic disease and 15.0% have multimorbidity. Of the participants, 6.9% were depressed, 7.7% were anxious, and 3.5% were stressed. A chi-square test demonstrated that depression, anxiety, stress, and chronic disease were significantly associated with the sleep quality of MEFC.

### 3.3. The Association between Chronic Disease, Mental Health, and Sleep Quality

Table 3 displayed the association between chronic disease, mental health, and sleep quality. Model 1 included sociodemographic variables that were statistically significant for sleep quality (sex, marital status, and monthly family income compared with others around). The results showed that sex, marital status, and monthly family income compared with others were important predictors of sleep quality among MEFC. When chronic disease entered into Model 2, sex, marital status, and monthly family income compared with others around remained significant, while the chronic disease was also significant. Model 3 added mental health based on Model 2; marital status has no statistical significance. Finally, the MEFCs who were male (OR = 0.334, 95%CI = 0.172–0.649) were less likely to report poor sleep quality, while the MEFCs with similar household income compared with others around (OR = 3.730, 95%CI = 1.089–12.780), multimorbidity (OR = 1.961, 95%CI = 1.079–3.564), depression (OR = 3.630, 95%CI = 1.547–8.519), and anxiety (OR = 2.321, 95%CI = 1.075–5.012) were more likely to report poor sleep quality.

Specifically, the OR value for MEFCs with multimorbidity was 1.961, meaning that those MEFCs who had multimorbidity were 1.961 times more likely than those without chronic disease to report poor sleep quality. The OR value of depression was 3.630, implying that those MEFCs who were depressed was 3.630 times than that without depression who reported poor sleep quality. The OR value of anxiety was 2.321, indicating that the MEFC who had anxiety were 2.321 times more likely than those without anxiety to report poor sleep quality.

## 4. Discussion

To the best of our knowledge, this is the first study to investigate the sleep quality of MEFC in Weifang City, Shandong Province, China. Moreover, this study also initially explored the relationship between chronic disease, mental health, and sleep quality, which could improve sleep quality among MEFC by providing some empirical evidence to health workers and governors, as well as those sleep quality-related researchers.

### 4.1. Sleep Quality Profile of MEFC in Weifang, China

In this study, 18.3% of MEFCs were defined as having poor sleep quality. This result is higher than that of a previous study, which found that the prevalence of poor sleep quality among the Chinese elderly people in Tianjin, China, was 14.39% [10]. One possible explanation for this difference is that adapting to a new environment in the city and fitting into a new community may put some mental pressure on their sleep. Meanwhile, the study’s result was lower than a study in China’s Henan Province that showed 40.1 percent of rural empty-nester older adults had poor sleep quality [47]. This could also be explained by the good living environment. The difference may come from living with family members, which may bring MEFC better living conditions and happiness [48], compared to empty-nester older adults. The prevalence of poor sleep quality was relatively low in this study compared with the 28.9% of Japanese elderly people who had poor sleep quality [15]. This difference may be explained by age disparity (the average age in the current study was 66.3 years, compared with 71.1 years in the Japanese study).

### 4.2. Association between Sociodemographic Variables and Sleep Quality

This study found that sex was associated with sleep quality in MEFC. Specifically, males were more willing to report good sleep quality than female MEFC. This was consistent with the previous studies showing that poor sleep quality was more prevalent in women [20,49]. In addition, Chinese rural elderly women had poorer sleep quality [50]. The possible reason for this phenomenon was that women were more likely to report mental disorders (for example, depression, anxiety, and stress) while 73.1% of MEFC were female in this study. The previous studies have demonstrated the association between mental health and sleep quality, which will be discussed in the next.

In addition, our findings also suggested that respondents with a similar level of household income were more likely to report poor sleep quality than those with higher monthly household incomes. One possible reason is that family income is the basis of daily life, and poor economic conditions may cause older adults to worry about the quality of life in a family, which in turn leads to psychological insomnia [51,52]. In this study, MEFC’s main living expense source was from their spouse and children (64.1%), which indicated their economic dependence on family members.

In terms of marital status, MEFCs who were married were more likely to report good sleep quality than those without a spouse, which was consistent with the previous study [53], although it was not significant in the last model. A possible reason was that loneliness caused by sleeping alone increases the stress hormone cortisol, leading to disrupted sleep and poorer sleep quality [54].

### 4.3. Association between Chronic Disease and Sleep Quality

Chronic diseases were found to be negatively associated with sleep quality in MEFC. Specifically, our results showed that MEFCs with multimorbidity reported worse sleep quality. In rural China, older people with chronic diseases were found to be more likely to suffer from poor sleep [55]. In previous studies, among older Hispanic adults in the United States [56] and Singapore [57], older adults with chronic diseases were more likely to have sleep problems, which was consistent with our findings. Existing studies pointed out that people with chronic diseases may experience pain, and the pain was negatively associated with sleep [58,59]. Moreover, older individuals with chronic conditions that require regular medication may experience dry mouth at night after taking medication. The need to drink more water may lead to an increase in the number of awakenings during the night and ultimately poorer sleep quality [60].

### 4.4. Association between Mental Health and Sleep Quality

This research also revealed that both depression and anxiety were negatively related to the sleep quality of MEFC. The results of this study were similar to previous findings that depression and anxiety were risk factors for poor sleep quality among community-dwelling older adults [61]. MEFC with depression and anxiety tended to report a higher incidence of poor sleep quality. Previous research conducted in South Korea [62] and Portugal [63] both demonstrated that depression was related to poor sleep quality among older adults. Moreover, Zhang et al. found that older adults with anxiety were more likely to have poor sleep quality in Beijing, China [64]. These results above were all consistent with our research findings.

### 4.5. Implications

This study aimed to comprehensively explore the effects of chronic disease and mental health on sleep quality among the MEFC in China and further provide useful theoretical and practical implications for improving the health of older people. Firstly, the findings that being depressed and anxious were risk factors affecting the sleep quality of MEFC suggest that regular monitoring of mental health is necessary to improve the sleep quality of MEFC. Thus, it is recommended that community health service centers and health professionals pay more attention to the mental health status of both community residents and MEFC. Secondly, multimorbidity was another predictable factor for MEFC, indicating the importance of chronic disease management. Information and education are needed in the control of chronic diseases among the MEFC. Besides, for MEFC without chronic diseases, more health interventions and knowledge programs about healthy living habits should be conducted to prevent the development of chronic diseases.

### 4.6. Limitations

The study has several limitations. Firstly, this study is a cross-sectional study, therefore the causal relationship between chronic disease, mental health, and sleep quality cannot be predicted. Secondly, the data of this study came from Weifang City, which has some limitations, and the representativeness of the results needs to be further improved for other regions in China. Thirdly, this study collected data by self-reporting so it may have a bias on recalling and reporting.

## 5. Conclusions

In conclusion, this is the first study that has clarified the effects of chronic disease and mental health on sleep quality among the MEFC population in China. The study pointed out that 18.3% of MEFCs had poor sleep quality, while the MEFCs with multimorbidity, depression, and anxiety were more likely to have poor sleep quality. It is expected that the findings of this research would provide an experiential contribution to improving sleep quality among MEFCs.

## Figures and Tables

**Table 1:** The sociodemographic characteristics of the MEFC.

Variables	N (%)	Sleep Quality		χ^2^	*p*
		Poor	Good		
Total	613 (100.0)	112 (18.3)	501 (81.7)		
Age (years)				3.847	0.146
60–69	486 (79.3)	85 (17.5)	401 (82.5)		
70–79	103 (16.8)	19 (18.4)	84 (81.6)		
80+	24 (3.9)	8 (33.3)	16 (66.7)		
Sex				12.741	<0.001
Male	165 (26.9)	15 (9.1)	150 (90.9)		
Female	448 (73.1)	97 (21.7)	351 (78.3)		
Hukou				0.384	0.536
Rural	525 (85.6)	98 (18.7)	427 (81.3)		
Urban	88 (14.4)	14 (15.9)	74 (84.1)		
Working Status				3.595	0.166
Employment	53 (8.6)	9 (17.0)	44 (83.0)		
Retired	126 (20.6)	16 (12.7)	110 (87.3)		
Unemployment	434 (70.8)	87 (20.0)	347 (80.0)		
Migration Willing				4.472	0.107
Unwilling	56 (9.1)	16 (28.6)	40 (71.4)		
Fair	45 (7.3)	7 (15.6)	38 (84.4)		
Willing	512 (83.5)	89 (17.4)	423 (82.6)		
Migration Reason				0.252	0.616
Taking care of grandchildren	533 (86.9)	99 (18.6)	434 (81.4)		
Not taking care of grandchildren	80 (13.1)	13 (16.3)	67 (83.8)		
BMI				2.974	0.390
Underweight	12 (2.0)	2 (16.7)	10 (83.3)		
Normal	241 (39.3)	52 (21.6)	189 (78.4)		
Overweight	290 (47.3)	48 (16.6)	242 (83.4)		
Obesity	70 (11.4)	10 (14.3)	60 (85.7)		
Marital Status				7.400	0.007
Married	539 (87.9)	90 (16.7)	449 (83.3)		
Single	74 (12.1)	22 (29.7)	52 (70.3)		
Education Level				1.384	0.709
Illiterate	161 (26.3)	34 (21.1)	127 (78.9)		
Primary school	185 (30.2)	31 (16.8)	154 (83.2)		
Junior high school	158 (25.8)	29 (18.4)	129 (81.6)		
High school and above	109 (17.8)	18 (16.5)	91 (83.5)		
Source of Living Expenses				8.089	0.015
Own	214 (34.9)	29 (13.6)	185 (86.4)		
Spouse and children	393 (64.1)	80 (20.4)	313 (79.6)		
Others	6 (1.0)	3 (50.0)	3 (50.0)		
Monthly Family Income Compared with Others Around				18.690	<0.001
Lower	282 (46.0)	69 (24.5)	213 (75.5)		
Fair	265 (43.2)	40 (15.1)	225 (84.9)		
Higher	66 (10.8)	3 (4.5)	63 (95.5)		

**Table 2:** Chronic disease, mental health, and sleep quality of MEFC.

Variables		N (%)	Sleep Quality		χ^2^	*p*
			Poor	Good		
Total		613 (100.0)	112 (18.3)	501 (81.7)		
Mental Health	Depression				30.399	<0.001
	Yes	42 (6.9)	21 (50.0)	21 (50.0)		
	No	571 (93.1)	91 (15.9)	480 (84.1)		
	Anxiety				32.056	<0.001
	Yes	47 (7.7)	23 (48.9)	24 (51.1)		
	No	566 (92.3)	89 (15.7)	477 (84.3)		
	Stress				16.943	<0.001
	Yes	21 (3.4)	11 (52.4)	10 (47.6)		
	No	592 (96.6)	101 (17.1)	491 (82.9)		
Chronic Diseases	Chronic Diseases	592 (96.6)	101 (17.1)	491 (82.9)		
	Zero				8.302	0.016
	One chronic disease	351 (57.3)	52 (14.8)	299 (85.2)		
	Multimorbidity	170 (27.7)	35 (20.6)	135 (70.4)		

**Table 3:** Logistic regression analysis on the association between sociodemographic characteristics, chronic disease, mental health, and sleep quality among MEFC.

Variable	Model 1			Model 2			Model 3		
	Sociodemographic			Model 1 + Chronic Disease			Model 2 + Mental Health		
	OR	95%CI	*p*	OR	95%CI	*p*	OR	95%CI	*p*
Sex									
Female	1.0			1.0			1.0		
Male	0.424	0.230–0.782	0.006	0.395	0.213–0.734	0.003	0.334	0.172–0.649	0.001
Marital Status									
Single	1.0			1.0			1.0		
Married	0.534	0.304–0.937	0.029	0.561	0.318–0.989	0.046	0.638	0.350–1.164	0.143
Source of Living Expense									
Others	1.0			1.0			1.0		
Spouse and children	0.249	0.045–1.366	0.109	0.242	0.044–1.330	0.103	0.277	0.047–1.650	0.159
Own	0.246	0.046–1.305	0.099	0.245	0.046–1.300	0.098	0.308	0.054–1.762	0.186
Monthly Family Income Compared with Others Around									
Higher	1.0			1.0			1.0		
Similar	5.315	1.547–17.942	0.007	5.162	1.520–17.532	0.009	3.730	1.089–12.780	0.036
Lower	3.205	0.945–10.872	0.062	3.213	0.943–10.949	0.062	2.601	0.759–8.913	0.128
Chronic Diseases									
Zero				1.0			1.0		
One chronic disease				1.618	01.990–2.645	0.055	1.321	0.788–2.215	0.291
Multimorbidity				2.158	1.219–3.820	0.008	1.961	1.079–3.564	0.027
Depression									
No							1.0		
Yes							3.630	1.547–8.519	0.003
Anxiety									
No							1.0		
Yes							2.321	1.075–5.012	0.032
Stress									
No							1.0		
Yes							1.274	0.398–4.083	0.683

## Data Availability

The datasets used and analyzed in this study are available from the corresponding author upon reasonable request.
